# Promoting and Maintaining Changes in Smoking Behavior for Patients Following Discharge from a Smoke-free Mental Health Inpatient Stay: Development of a Complex Intervention Using the Behavior Change Wheel

**DOI:** 10.1093/ntr/ntac242

**Published:** 2022-10-17

**Authors:** Emily Shoesmith, Lisa Huddlestone, Jodi Pervin, Lion Shahab, Peter Coventry, Tim Coleman, Fabiana Lorencatto, Simon Gilbody, Moira Leahy, Michelle Horspool, Claire Paul, Lesley Colley, Simon Hough, Phil Hough, Elena Ratschen

**Affiliations:** Department of Health Sciences, University of York, Heslington, York, UK; Department of Health Sciences, University of York, Heslington, York, UK; Department of Health Sciences, University of York, Heslington, York, UK; Department of Behavioural Science and Health, University College London, London, UK; York Environmental Sustainability Institute, University of York, York, UK; Centre for Academic Primary Care, University of Nottingham, Nottingham, UK; Centre for Behaviour Change, University College London, London, UK; Department of Health Sciences, University of York, Heslington, York, UK; Hull York Medical School, University of York, York, UK; Research and Development, Sheffield Health and Social Care NHS Foundation Trust, Sheffield, UK; Research and Development, Sheffield Health and Social Care NHS Foundation Trust, Sheffield, UK; Research and Development, Leeds and York Partnership NHS Foundation Trust, Leeds, UK; Research and Development, Tees, Esk and Wear Valleys NHS Foundation Trust, Stockton on Tees, UK; Independent Peer Researcher, Cheshire, UK; Independent Peer Researcher, Cheshire, UK; Department of Health Sciences, University of York, Heslington, York, UK

## Abstract

**Introduction:**

Evidence suggests that smokers can successfully quit, remain abstinent or reduce smoking during a smoke-free mental health inpatient stay, provided behavioral/pharmacological support are offered. However, few evidence-based strategies to prevent the return to prehospital smoking behaviors post-discharge exist.

**Aims and Methods:**

We report the development of an intervention designed to support smoking-related behavior change following discharge from a smoke-free mental health stay. We followed the Behavior Change Wheel (BCW) intervention development process. The target behavior was supporting patients to change their smoking behaviors following discharge from a smoke-free mental health stay. Using systematic reviews, we identified the barriers and enablers, classified according to the Theoretical Domains Framework (TDF). Potential intervention functions to address key influences were identified by consulting the BCW and Behavior Change Technique (BCT) taxonomy. Another systematic review identified effectiveness of BCTs in this context. Stakeholder consultations were conducted to prioritize and refine intervention content.

**Results:**

Barriers and enablers to supporting smoking cessation were identified within the domains of environmental context and resources (lack of staff time); knowledge (ill-informed interactions about smoking); social influences, and intentions (lack of intention to deliver support). Potential strategies to address these influences included goal setting, problem-solving, feedback, social support, and information on health consequences. A strategy for operationalizing these techniques into intervention components was agreed upon: Pre-discharge evaluation sessions, a personalized resource folder, tailored behavioral and text message support post-discharge, and a peer interaction group, delivered by a trained mental health worker.

**Conclusions:**

The intervention includes targeted resources to support smoking-related behavior change in patients following discharge from a smoke-free mental health setting.

**Implications:**

Using the BCW and TDF supported a theoretically and empirically informed process to define and develop a tailored intervention that acknowledges barriers and enablers to supporting smoking cessation in mental health settings. The result is a novel complex theory- and evidence-based intervention that will be formally tested in a randomized controlled feasibility study.

## Introduction

Smoking remains a leading cause of mortality and morbidity worldwide.^1^ Smoking prevalence in the general population in England has steadily declined from 14.1% in 2019 to 13.5% in 2020,^2^ but data indicate that prevalence remains approximately two to three times higher among people with mental health conditions in the United Kingdom.^3^ Smokers with mental health conditions are more likely to experience greater dependence on smoking, are more likely to develop smoking-related illnesses, and long-term quit rates among this population are lower than for the general adult population.^3–5^ Although people with mental health conditions are just as motivated to quit as those in the general population,^,3,6^ they are less likely to receive the support they require compared with smokers without mental health conditions.^7^

Many healthcare settings are now smoke-free by law and offer smoking cessation support for smokers during their stay.^8^ Evidence suggests that individuals can successfully remain abstinent during their inpatient stay when behavioral/pharmacological support is offered.^9,10^ Additionally, evidence from mental health settings indicates that a period of supported abstinence may promote smoking behavior change and motivation to quit.^11^ However, the risk of relapse postdischarge is high, with one study reporting 76% relapsed to smoking behaviors within one-day postdischarge.^12^ Lack of support offered post-discharge and the high risk of relapse renders smoking-related resource input during the inpatient episode inefficient, as positive smoking behavior change achieved during the inpatient stay is often lost. Identifying interventions that are effective in supporting patients around the time of discharge in maintaining or achieving positive change to their smoking behaviors has been identified as an important evidence gap in this area.^13^

Designing interventions to support smoking cessation would benefit from drawing on theories and frameworks from behavioral science which summarize drivers of behaviors and link these to strategies for changing behavior.^14^ The Behavior Change Wheel (BCW)^15^ and Theoretical Domains Framework (TDF) are developments in the behavior change field that provide a systematic, theoretical basis for understanding and providing tools to change behavior,^16^ based on multiple models of health behavior. The process of intervention development using the BCW and TDF is outlined in detail^15^ and has been extensively applied to design smoking cessation interventions.^17–19^

### Theoretical Underpinnings

The BCW was developed from an extensive review of behavioral science frameworks, designed to facilitate the development of behavior change interventions.^15,20^ At the center of the BCW is a behavior change model known as the COM-B, which postulates that behavior change is essentially dependent on the interaction between three key determinants: The capability (C) to perform a behavior, the opportunity (O) and motivation (M) to perform.^15^ The TDF is an elaboration of the COM-B model and includes 14 domains based on an integration of behavioral theories.^21–23^ These domains prompt the consideration of a wide range of influences as they include individual-level factors, social factors, and environmental factors.^16^

The COM-B and TDF have been mapped to frameworks that specify different intervention strategies. The first is the BCW which specifies nine intervention types for changing behavior (e.g. education, training).^15^ These intervention functions are supported by seven policy categories, representing decision types that help to support the interventions (e.g. communication/marketing, guidelines).^15^ The second is the more granular taxonomy of Behavior Change Techniques (BCTs), which breaks down these broad intervention types into 93 discrete BCTs (e.g. goal setting, action planning, feedback on behavior).^23^ There are published tools that pair domains from the COM-B/TDF with intervention strategies in the BCW/BCT taxonomy (BCTv1)^23^ to specify which types of behavior change approaches are more likely to be relevant and effective in changing different influences on behavior.^15^ This provides a step-wise process from moving from an initial behavioral diagnosis to identifying “what needs to change,” to selecting specific intervention components in a theory- and evidence-informed manner.^16^

To the best of our knowledge, the BCW and TDF have not been applied to the context of smoking cessation following discharge from a smoke-free mental health inpatient stay. The aim of this study was to apply the BCW and TDF to systematically design evidence- and theory-based complex interventions for people with a mental health condition to support smoking-related behavior changes following a mental health inpatient stay. This process is in line with the Medical Research Council’s (MRC) guidance for developing complex interventions.^14^

## Method

### Aim

To develop an evidence- and theory-based complex intervention to support smoking-related behavior changes for patients following discharge from a smoke-free mental health inpatient stay.

### Research Design

**Figure 1.**
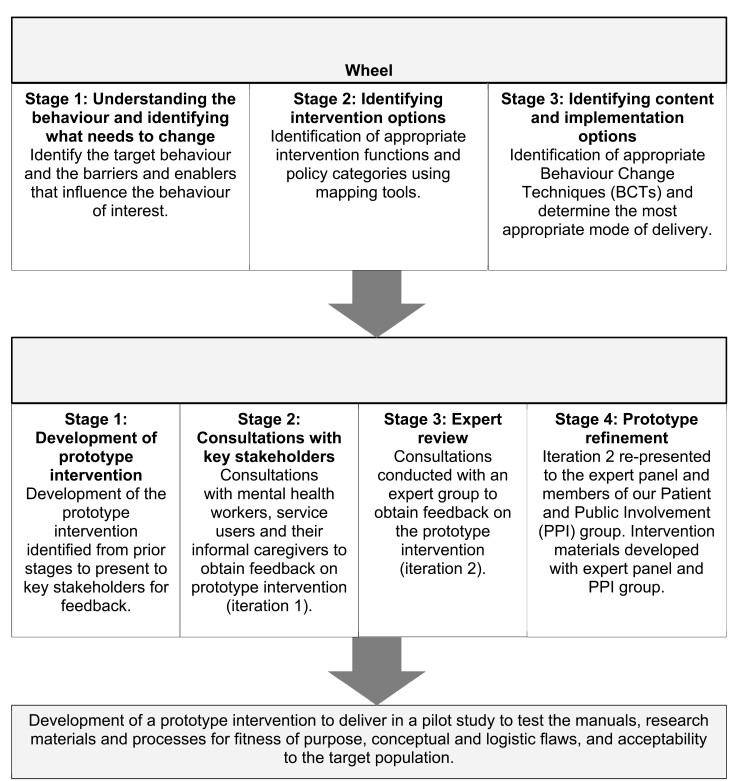
Intervention development process.

Intervention development involved two sequential phases: (1) synthesizing findings from existing evidence and mapping these onto the BCW, and (2) using qualitative and consensus methodologies to explore acceptability and feasibility of the prototype intervention (Figure 1).

### Phase 1: Content Development

The process of intervention development using the BCW and TDF is outlined in detail elsewhere,^15^ and a detailed explanation of how the process was followed is presented in Supplementary Material 1. A summary is provided:

#### Stage One: Understanding the Behavior and Identifying “What Needs to Change”

The BCW was used to understand the healthcare problem and the behaviors that could be targeted by an intervention.^15^

To identify what was influencing behaviors and the barriers to/enablers of change, we conducted a systematic review of the published qualitative and quantitative literature investigating smoking cessation interventions in mental health settings. We extracted and thematically synthesized barriers and enablers. These were then coded to the domains of the COM-B and TDF. The detailed methods are published elsewhere.^24^

#### Stage Two: Identifying Intervention Options

We mapped identified influences on potential intervention strategies using the aforementioned mapping tools which pair COM-B and TDF domains to the BCW and BCTs.^15^ This resulted in a long list of potentially relevant intervention functions. The APEASE criteria were used to guide judgments as to what functions may be the most appropriate for the intervention.^15^ To prioritize further, and identify what interventions have been done to date in this context, we also conducted a systematic review of behavioral and pharmacological interventions that maintain abstinence following a smoke-free stay and determined their effectiveness.^10^

We then mapped the identified intervention functions to potential policy categories that are likely to be appropriate and effective in supporting each function.^15^ The APEASE criteria were used to help prioritize amongst these potential categories.

#### Stage Three: Identifying Content and Implementation Options

We considered all BCTs that could be considered for any particular function, using the aforementioned mapping tools.^15^ This resulted in a long list of potentially appropriate BCTs. The APEASE criteria were used to select those most likely to be appropriate. To further guide selection, we coded existing intervention descriptions into component BCTs using the BCTTv1 and identified which BCTs were defined as “promising” in terms of probable effectiveness and feasibility. We have previously reported these methods in detail.^10^ We determined the most appropriate mode of delivery.

We selected potential intervention strategies and produced a description of a prototype intervention which was then presented to key stakeholders and an expert group of academics and clinicians (Supplementary Material 2; Table 2A).

### Phase 2: Iterative Refinement Based on Stakeholder and Expert Group Input

Consultations were conducted with stakeholders and an expert group of academics and clinicians to identify potential problems with the prototype intervention, develop solutions and assess the perceived acceptability, practicability, and ease of integration.

#### Stakeholder Consultations

##### Settings and Participants

Consultations were conducted with four North of England NHS Trusts. Stakeholders included: (1) users of mental health services, (2) informal caregivers, and (3) mental health workers in acute adult mental health inpatient and community mental health services. The term mental health worker (MHW) refers to any mental healthcare professional or healthcare associate role, and the worker did not have to be professionally qualified.

##### Recruitment

MHWs were invited by their service leads or through direct contact with the research team in liaison with Trusts. Patient and caregiver stakeholders were invited to volunteer through direct contact by the Trust Patient and Public Involvement (PPI) lead; by responding to advertisements in Trust newsletters.

##### Methods

Consultations took place remotely with individuals or groups. The prototype intervention was presented to stakeholders, and a guided discussion informed by the BCW elicited detailed feedback. Additionally, online presentation software was used during the MHW consultations. This software used 5-point Likert scales to obtain ratings for individual intervention components in relation to practicality, acceptability to staff, patients, and caregivers, and the perceived ease of integration into services. The 5-point Likert scale included response anchors at either end of the scale (1 = low; 5 = high).

##### Analysis

Intervention component ratings were summarized using descriptive statistics. Qualitative data from the guided discussions were exported to NVivo 12 software^25^ and analyzed using thematic analysis,^26^ employing an inductive approach, in which coding and theme development were driven by the content of the responses. One author (E.S) familiarized herself with the data and notes were made of any potential codes by identifying recurring words or units of meaning. The same author generated initial codes from the data and organized them into meaningful groups. Codes were then organized into potential themes and all relevant coded responses were collated within the identified themes. Three authors (E.S, L.H, and J.P) independently reviewed the construction of themes and relevant quotations to agree to the assignment of themes. Inductive thematic analysis was used to identify and categorize potential barriers and enablers in relation to the development and implementation of the intervention, which was subsequently mapped to the COM-B model and facilitated further intervention refinement. The findings from this process were used to refine the prototype intervention (Supplementary Material 2; Table 2B).

#### Expert Advisory Group Assessment of Prototype Intervention

The prototype intervention was presented during expert panel consensus meetings. The expert panel involved involving academic experts in tobacco control and intervention design (*n* = 13), and clinicians from participating Trusts (*n* = 4). All experts in the advisory group had been approached to be collaborators/advisors or co-applicants for the research before it commenced, and all accepted and provided feedback. Feedback was elicited through discussion and topics related to overall perceptions, delivery pathways, and potential implementation issues. During the meetings, notes of key points were taken, and written summaries of each meeting were shared with the expert panel to ensure they were accurate overviews of the discussions. Final summaries were discussed in detail to support the refinement of the intervention. The feedback from this process was used to further refine the prototype intervention (Supplementary Material 2; Table 2C).

#### Expert Advisory Group and PPI Group Final Review

Following stakeholder consultations and expert group meetings, the revised prototype was re-presented to the expert advisory panel for final feedback. The revised prototype was also presented to members of the research program PPI group. This PPI group was formed following the stakeholder consultations and includes current and ex-smokers who have experienced an inpatient mental health admission. The materials required for the intervention were developed and reviewed by our PPI group and an expert panel, to ensure these materials were appropriately worded, engaging, and ease to use.

## Results

### Phase 1: Content Development

#### Stage One: Understanding the Behavior and Identifying What Needs to Change

The target behavior of interest was defined as: Adult smokers in mental health settings changing their smoking-related behaviors following discharge from a smoke-free mental health inpatient stay.

The findings of the systematic review to identify the barriers and enablers to influence the behavior of interest have been published elsewhere.^24^ In summary, the key barriers to patients making smoking-related behavior changes in mental health settings fell within the following TDF domains: Environmental context and resources (e.g. lack of staff time); knowledge (e.g. interactions were ill-informed); social influences (e.g. smoking norms), and intentions (e.g. lack of positive intentions). Key enablers mainly fell within the domains: Environmental context and resources (e.g. use of appropriate support materials) and social influences (e.g. pro-quitting social norms) (Supplementary Material 3).

#### Stage Two: Identifying Intervention Options

The COM-B/TDF domains were mapped to the nine intervention functions identified within the BCW (Supplementary Material 3). The intervention functions of education, training, and enablement were included, based upon an evaluation of their affordability, practicability, effectiveness, acceptability, side-effects and safety, and equity (APEASE).^15^ Details of each intervention function against the APEASE criteria are provided in Supplementary Material 4; Table 4a.

The intervention functions were then mapped to the policy categories identified within the BCW (Supplementary Material 3). The policy categories of communications/marketing, guidelines, and service provision were included, based on an evaluation of the APEASE criteria. Details of the evaluation of each policy category against the APEASE criteria are provided in Supplementary Material 4; Table 4B.

#### Stage Three: Identifying Content and Implementation Options

The intervention functions identified were then mapped to a potentially long list of BCTs using the BCTTv1^15^ (Supplementary Material 3). The APEASE criteria and findings from the second systematic review^10^ were used to guide the identification of the most appropriate BCTs to use within the intervention (Supplementary Material 5). The full findings of the systematic review have been published elsewhere.^10^ In summary, nine BCTs (including “pharmacological support,” “goal setting (behavior),” and “social support”) were characterized as “promising” in terms of probable effectiveness and feasibility. A review of the full BCTTv1 identified additional BCTs: “social award,” “self-reward,” “pharmacological support,” and “restructuring the social environment,” which also met the APEASE criteria.

A blended approach encompassing face-to-face and remote delivery was chosen, as the predischarge evaluation session and provision of resources is planned to occur on the inpatient ward predischarge. Postdischarge, the intervention will be delivered remotely, including behavioral support calls via telephone/video call and optional text message support. This approach was considered in terms of the evidence in relation to remote interventions and in the context of Covid-19-related restrictions. Both delivery modes met the APEASE criteria.

A prototype intervention strategy was drafted (Supplementary Material 6), based on the existing evidence and selections made throughout the BCW process.

### Phase 2: Iterative Refinement Based on Stakeholder and Expert Group Input

#### Stakeholder Consultations

Twenty-five stakeholders participated to assess the practicality, acceptability, and ease of integration of the prototype intervention. Key stakeholders included MHWs, patients with mental health conditions, and one informal caregiver (Table 1).

**Table 1.** Stakeholder demographics (*n* = 25)

Mental health workers (*n* = 17)		*N* (%)
*Gender*	Female	10 (58.8)
*Setting*	Inpatient	11 (64.7)
	Community	6 (35.3)
*Job role*	Nurse	8 (47.0)
	Healthcare assistant	3 (17.6)
	Occupational therapist	1 (5.9)
	Unit manager	1 (5.9)
	Smoking cessation advisor	2 (11.8)
	Healthy living advisor	2 (11.8)
Patients (*n* = 7)		***N* (%)**
*Gender*	Female	2 (28.6)
*Smoking status*	Recently quit smoking	4 (57.1)
*Admission/discharge status*	Currently using acute inpatient service	2 (28.6)
	Community-based, and have had previous experience of using acute inpatient services	5 (71.4)
Caregiver (*n* = 1)		***N* (%)**
*Gender*	Female	1 (100)
*Smoking status*	Never smoker	1 (100)

The prototype intervention (Supplementary Material 2; Table 2A) was well-received, and most individual components were rated highly for practicality, acceptability, and ease of integration (mean scores ranging between 4 and 4.8). The identification of a “buddy” to provide social support postdischarge. This component had a lower score in terms of practicality, acceptability, and ease of integration compared to other components (mean scores ranged between 3.2 and 4.6) and alternative components were explored. The MHWs ratings for individual intervention components are provided in Supplementary Material 7.

Overall, four themes were identified from the guided discussion with stakeholders (Supplementary Material 8). The largest number of barriers for MHWs were classified under physical opportunity, including beliefs about limited staff time and resources. From a patient and caregiver perspective, barriers were classified under reflexive motivation, which included beliefs about MHWs not holding positive intentions to support smoking cessation.

#### Theme One: Enablers of Patient Engagement and Intervention Delivery

In relation to current support, many MHWs identified the absence of a strong link between the inpatient ward and the community. They highlighted that to provide effective support post-discharge, there needed to be improved communication between inpatient/community teams and a seamless process of transferring patient information. For many patients, starting their journey during their stay and continuing this progress into the community was perceived as an important factor in their motivation to continue engaging with smoking cessation support. Patients expressed the importance of this continuum, as opposed to inpatient support and “separate” community support. There was a consensus that the intervention would facilitate a smoother transition from inpatient to community and would enhance patient engagement. All MHWs acknowledged that the predischarge assessment was critical, and it was important that the information collated in this session had to be fed into the community to avoid duplication. All stakeholders believed providing the resource pack to patients predischarge would “bridge the gap” between inpatient and community support, as patient information would be in one place from inpatient to community. All stakeholders also reported that pre-discharge provision was advantageous as the patients would have a plan in place predischarge that would continue in the community.

All stakeholders believed that setting personalized goals and the provision of a resource pack at discharge would be beneficial. All stakeholders believed the motivational and practical content included in the pack would facilitate participant engagement. Finally, remote support postdischarge was frequently met with approval, as receiving remote behavioral support calls quickly and easily would facilitate accessibility. It was important to all stakeholders that the behavioral support was delivered by the same MHW to build a good relationship with the patient.

#### Theme Two: Potential Barriers to Intervention Delivery

MHWs frequently mentioned that capacity and resources may be limited, as staff might often lack the time or opportunity to deliver support to patients alongside their existing workload. Additionally, there was a consensus across stakeholder groups that staff was often resistant to the idea of engaging in smoking cessation support, especially when staff members smoked themselves.

#### Theme Three: Overall Perceptions

Most MHWs agreed that the pre-discharge session designed for goal setting should be a critical component of smoking cessation support. This session is designed to tap into an individual’s wishes and needs to offer patients the most appropriate and tailored support. Patients also indicated the importance of flexibility within the goal-setting session as every individual would have unique goals and motivations that would require regular review.

Stakeholders believed that the resource pack was an innovative and novel idea that would be well-received by patients, as patients are provided with little written information. MHWs felt that including resources on the benefits of quitting within the pack would be advantageous. Telephone behavioral support was identified as an important, essential component by all stakeholders. Many stakeholders believed this would be well-received if the support was individualized. Finally, the text message component was well-received by all stakeholders, with most individuals suggesting these would maintain motivation during a quit attempt.

#### Theme Four: Practical Considerations and Suggestions

The prototype intervention included a “buddy” who could offer social support to individuals. However, most stakeholders highlighted that patients may not be able to identify a relative or friend who is able or willing to adopt this role. All stakeholders suggested it would be beneficial to replace this component with the formation of a social support group (Supplementary Material 2; Table 2B). By offering this opportunity, stakeholders believed this would enhance the support received from others experiencing a similar situation in an informal, supportive environment. One patient suggested that this group should be organized to encourage peer-to-peer support but have MHW present to facilitate interactions.

For the resource pack, most stakeholders suggested that the inclusion of “success stories” which illustrated positive behavior changes made by others in similar situations would provide motivational support. Two patients suggested additional content could be included to outline alternative activities that may offer a distraction (Supplementary Material 2; Table 2B). It was noted this information should be presented in a creative, user-friendly manner. All stakeholders were positive about the acceptability and feasibility of using motivational text messages, but with the option to opt-in or -out should they be seen as overwhelming (Supplementary Material 2; Table 2B).

#### Expert Advisory Group Assessment of Prototype Intervention

The expert advisory group perceived the intervention to be acceptable and feasible but highlighted that it would be critical to identify an appropriate person to deliver it (Supplementary Material; Table 2C). Group members acknowledged that when identifying MHW to deliver the intervention, this should not be restricted by role, but rather those who fulfill a certain criterion. For example, experience in working with mental health and have a positive outlook on supporting people with mental health conditions to change their smoking-related behaviors. It was highlighted that there is no evidence to suggest certain job roles are better than others in terms of their effectiveness in helping individuals to positively change their smoking behaviors. The expert group indicated that it would not be advisable to exclude potential facilitators due to their job titles when they may be experienced and willing to deliver the intervention effectively. A document was created in collaboration with the expert panel to provide an overview of the MHW who would deliver the intervention, outlining the role description, person specification, and training requirements.

In the systematic review, “pros and cons” and “framing/reframing” were identified as “promising” in terms of likely effectiveness, but not in terms of likely feasibility.^10^ Clinicians from the expert group discussed the importance of including these BCTs, as they appeared particularly important in a mental health inpatient context to help the individual reflect on their smoke-free experience and explore their motivation to remain smoke-free or positively change their smoking behavior. During the predischarge evaluation, the MHW could provide motivational enhancement and strategies for managing temptations, considering the pros and cons of change. Additionally, the patient could be presented with alternative ways of thinking and counterarguments to their belief barriers and discuss behavioral self-management strategies to counter triggers.

The expert group also discussed the opt-in/-out process for the text-messaging component that was included in line with the feedback from the stakeholder consultations. The expert group acknowledged the decision to make this component optional but highlighted how text-messaging support has been found to be effective for smoking cessation. Therefore, they suggested that participants should be automatically enrolled in this component unless they express a negative preference during the pre-discharge evaluation. Participants should also be able to opt out of the component at any time by replying “STOP” to any of the messages or by contacting the individual delivering the intervention (Supplementary Material; Table 2C).

### The Final Prototype Intervention

The development process of identifying key domains through intervention content is presented in Supplementary Material 9. The final prototype intervention consists of components that aim to support smoking-related behavior change among patients following discharge from a smoke-free mental health inpatient setting. Intervention components include: (1) pre-discharge reflection and evaluation, (2) a personalized resource folder (My Try folder), (3) tailored behavioral support (via telephone or video call), (4) optional text-messaging support, and (5) optional opportunity for peer interaction (Supplementary Material 10). The intervention will be delivered by a trained MHW (named the “My Try Specialist,” to link with the name of the personalized resource folder). The name “My Try” was discussed and agreed upon with the stakeholders, expert group, and PPI group, and was identified as appropriate as it indicated flexibility dependent on individual patient motivations.

Supplementary Material 11 provides a detailed description of the intervention using the 12-item Template for Intervention Description and Replication (TIDieR) checklist4.^27^

## Discussion

The intervention consists of five components that together aim to support smoking-related behavior change among patients following discharge from a smoke-free mental health inpatient setting. This intervention draws on behavioral science to specifically target the barriers/enablers to supporting smoking cessation in mental health settings and draws on existing evidence. A large body of literature recognizes the challenges that people with mental health conditions experience when making smoking-related behavior changes, including low self-efficacy,^28,29^ smoking norms,^24,30^ widely held misconceptions about the links between mental health and smoking,^31,32^ and a lack of support offered compared to that available to the general population.^33^ Therefore, it is important that interventions delivered to people with mental health conditions provide flexible support tailored to individual needs and are delivered by MHWs who are experienced in assisting people with mental health conditions.^34,35^ By examining the literature, the current prototype incorporates content that has been reported as successful for this population group,^10^ while addressing commonly cited barriers.^24^ Stakeholder consultations ensured that the prototype intervention met the target population’s needs and preferences. This in-depth approach to intervention development increases the likelihood that it will achieve smoking-related behavior change post-discharge. The next stage is to deliver the prototype intervention in a small-scale pilot study to test the intervention and processes for the fitness of the purpose. A process evaluation will highlight the need for further refinements. Subsequently, the intervention will be tested in a randomized controlled feasibility study.

The prototype intervention includes several remote components. Literature indicates that telephone support increases the chances of stopping smoking, whether the individuals are motivated to quit or not, or if they are receiving other support.^36^ The COM-B model supports remote smoking cessation treatment as this approach may provide the opportunity to access support by removing barriers (e.g., travel and cost), particularly for those where accessing face-to-face services may be challenging.^37,38^ Text messaging is also an established cessation modality^39^ and has been shown to promote smoking cessation in the short term^40^ and long term.^41^ The opportunity for a remote peer interaction group was selected as a component to replace the identification of a support “buddy.” There is strong evidence to suggest that social networks play an important role in an individual’s quit attempt,^42,43^ and we found all “social support” BCTs were identified as “promising” both in terms of likely effectiveness and feasibility.^10^ Therefore, it was important to include an intervention component that aims to enhance social support, and evidence suggests remote social support can enhance smoking cessation outcomes.^44^

Although the remote support was well-received by the stakeholders, it is important to consider this mode of delivery may present challenges if participants have restricted access to technology and/or limited IT skills. Research has demonstrated that people with mental health conditions are at an increased risk of digital exclusion.^45,46^ However, this was not identified as a potential barrier during our consultations, and the increasing use of remote communication to provide health services during the Covid-19 pandemic illustrates the advantages of remote counseling.^47^

### Limitations

During stakeholder consultations, we collected patient and caregiver demographics including gender and smoking status. However, we did not collect demographics such as ethnicity and socioeconomic status, which would have further helped to contextualize the findings. Secondly, informal caregivers are under-represented in the stakeholder consultations. Recruitment of informal caregivers would have provided a valuable perspective for those who have supported relatives or friends who are current or ex-smokers and have experience using acute inpatient services. However, recruiting informal caregivers of smokers with a mental health condition is acknowledged as difficult,^24^ and consultations were conducted at the beginning of the pandemic so recruitment was conducted remotely, restricting the opportunity to meet with potential caregivers in inpatient wards. This highlights the need for further investigation into the role of informal support networks, and the development of targeted strategies to recruit informal caregivers.

## Conclusions

People with mental health conditions are more likely to smoke than the general population, which contributes to widening tobacco-related health inequalities. This intervention aims to support smoking-related behavior change in patients discharged from a smoke-free mental health inpatient stay, encompassing components that have been guided by existing evidence and appropriate BCTs. It includes resources and approaches that have been developed in collaboration with stakeholders to ensure they appeal to patients with mental health conditions following a smoke-free stay. Using the BCW and TDF supported a theoretically and empirically informed process to define and develop a tailored intervention that acknowledges barriers and enablers to supporting smoking cessation in mental health settings. The result is a novel complex theory- and evidence-based intervention that will be formally tested in a randomized controlled feasibility study.

## Supplementary Material

A Contributorship Form detailing each author’s specific involvement with this content, as well as any supplementary data, are available online at https://academic.oup.com/ntr.

ntac242_suppl_Supplementary_Material

ntac242_suppl_Supplementary_Taxonomy-form

## Data Availability

Data is available at: https://osf.io/jdw9y/.
