# Supplementary material for: Promoting and Maintaining Changes in Smoking Behavior for Patients Following Discharge from a Smoke-free Mental Health Inpatient Stay: Development of a Complex Intervention Using the Behavior Change Wheel
Source: Nicotine Tob Res. 2022 Oct 17;25(4):729–37. doi: 10.1093/ntr/ntac242 (PMC10032184; doi:10.1093/ntr/ntac242)
Supplement: ntac242_suppl_Supplementary_Material [file ntac242_suppl_supplementary_material.docx]

Supplementary Material

[Supplementary Material 1. Development phase for content (Behaviour Change Wheel) 2](#_Toc115359248)

[Supplementary Material 2. Prototype intervention and refinements 5](#_Toc115359249)

[Table 2a. Prototype intervention developed from existing evidence and BCW process 5](#_Toc115359250)

[Table 2b. Feedback from key stakeholders and refinements to prototype intervention 7](#_Toc115359251)

[Table 2c. Feedback from expert group and further refinements to prototype intervention 10](#_Toc115359252)

[Supplementary Material 3. Findings from the behavioural analysis mapped onto the BCW. 13](#_Toc115359253)

[Supplementary Material 4. APEASE criteria for intervention functions and policy categories 23](#_Toc115359254)

[Supplementary Table 4a. Selection of intervention functions 23](#_Toc115359255)

[Supplementary Table 4b. Selection of policy categories to support intervention delivery 23](#_Toc115359256)

[Supplementary Material 5. Selected BCTs for the prototype intervention and their definition 24](#_Toc115359257)

[Supplementary Material 6. Prototype intervention strategy 26](#_Toc115359258)

[Supplementary Material 7. Mental healthcare workers mean scores, medians, ranges, and interquartile ranges (IQRs) for each intervention component in terms of practicality, acceptability, and ease of integration. 28](#_Toc115359259)

[Supplementary Material 8. Example quotations for themes 30](#_Toc115359260)

[Supplementary Material 9. Final prototype intervention mapping table 33](#_Toc115359261)

[Supplementary Material 10. Prototype intervention specification 38](#_Toc115359262)

[Supplementary Material 11. Description of the prototype intervention using the TIDieR checklist 39](#_Toc115359263)

[Supplementary Material 12. Example text message content for participants 42](#_Toc115359264)

[Supplementary Table 12a. Text message content – Quit attempt example 42](#_Toc115359265)

[Supplementary Table 12b. Example text message content – Not yet ready to quit example 44](#_Toc115359266)

**Supplementary Material 1.** Development phase for content (Behaviour Change Wheel)

The Behaviour Change Wheel (BCW) process of intervention development involves three key stages: (1) understanding the behaviour and identifying what needs to change, (2) identifying intervention functions, and (3) identifying content and the intervention mode of delivery (see Figure 1).

**Figure 1.** Stages and steps required to develop and implement an intervention from the BCW (Michie, Atkins & West, 2014).


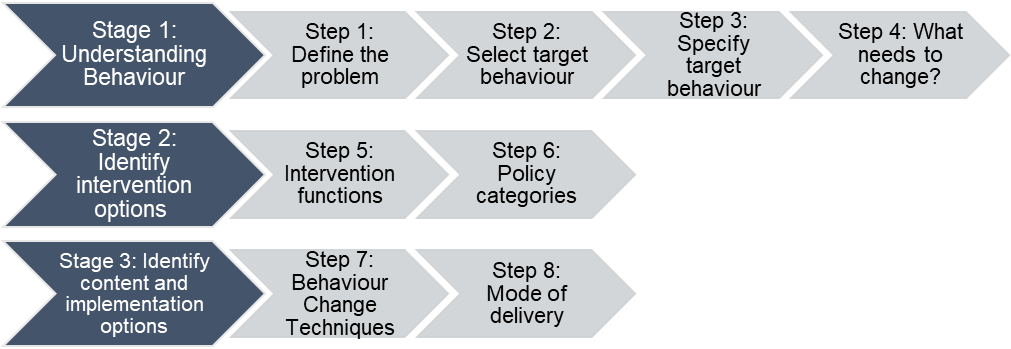


Stage 1: Understand the behaviour and identifying what needs to change

*Step 1: Defining the problem in behavioural terms*

This step involved defining the problem in behavioural terms, being specific about the population involved in the behaviour, and the behaviour itself.

*Step 2: Selecting the target behaviour*

This step involved considering the possible behaviours that could be targeted in interventions to support patients to remain smokefree post-discharge and prevent relapse to smoking or, where they have not been abstinent during admission, to achieve positive change to their smoking-related behaviours. To explore possible target behaviours, four members of the research team (ES, LH, JP, ER) held extensive discussions, guided by existing evidence to explore possible target behaviours.

*Step 3: Specifying the target behaviour*

This step involved detailing the specifics of the behaviour, frequency, duration, and the context in which it needs to occur in order for the behaviour to be carried out.

*Step 4: Identifying what needs to change*

After specifying the target behaviour, the next step is to identify what needs to change. The COM-B model is the starting point used by the BCW for understanding behaviour in the context in which it occurs. Data from the first systematic review were subject to behavioural analysis, as outlined by the BCW framework. The TDF was used to expand on COM-B components identified in the behavioural diagnosis.

Stage 2: Identify intervention options

*Step 5: Identifying appropriate intervention functions*

The BCW facilitates the identification of intervention functions, defined as broad categories of means by which an intervention or its component can change behaviour. The nine intervention functions are provided in the BCW and are linked with the COM-B components and TDF domains. Three members of the research team (ES, LH, JP) considered each of the candidate intervention functions using the APEASE criteria. The APEASE criteria as defined in the BCW (affordability, practicability, effectiveness and cost-effectiveness, acceptability, side-effects and safety, equity) were considered to direct the selection of appropriate intervention options. To further help prioritise amongst these and identify what interventions have been done to date in this context, we also conducted a systematic review of behavioural and/or pharmacological interventions that maintain abstinence following a smokefree stay and determined their effectiveness.

*Step 6: Identifying policy categories*

The BCW outlines seven policy categories, representing types of decisions made by authorities that help to support and enact the interventions. The BCW suggests which policy categories are likely to be appropriate and effective in supporting each intervention function (see step 5). Three members of the research team selected each of the policy categories based on consideration of the APEASE criteria.

Stage 3: Identify content and implementation options

*Step 7: Identifying Behaviour Change Techniques (BCTs)*

Three members of the research team (ES, LH, JR) independently generated a list of all the possible BCTs aligned with the selected intervention functions (see step 5). Through a process of discussion (and using the APEASE criteria), three members of the research team considered which BCTs could most feasibly be applied within the context of a post-discharge intervention and would be most useful for addressing the identified barriers to the target behaviours. To further guide the identification of appropriate BCTs, the findings from the second systematic review identified ‘promising’ BCTs in terms of likely effectiveness, and ‘promising’ BCTs in terms of both likely effectiveness and feasibility.

*Step 8: Determining the mode of delivery*

To select the preferred modes of delivery, three members of the research team held discussions with academic experts in tobacco control and intervention design, and clinicians from participating Trusts, while considering the APEASE criteria.

# **Supplementary Material 2.** Prototype intervention and refinements

| Table 2a. Prototype intervention developed from existing evidence and BCW process | | |
| --- | --- | --- |
| Prototype intervention components | **Aim of component** | **Summary of evidence** |
| Pre-discharge components | | |
| Pre-discharge assessment and flexible goal setting | This assessment will ensure the participants are aware of the benefits of continuing efforts to change their smoking behaviours. The session will provide personalised support and tailored goal setting, strategy, and contingency planning to assist participants to continue tobacco related changes post-discharge. | Effective interventions identified in existing evidence offered continuous reviewing of goals. BCTs in the ‘goals and planning’ cluster were identified as promising in terms of likely effectiveness and feasibility in our second systematic review. |
| Patient resource pack provided by clinician at discharge | Provide the participant with a personalised folder to assist them with achieving their behavioural change goals. A resource folder will contain journal pages to promote reflection and monitoring of progress, general information on the health benefits of quitting smoking, money saved information sheet, and tips on how to make a smokefree home. | Effective interventions in systematic review provided educational/self-help literature for patients during their stay and/or at point of discharge. Evidence suggests information distributed by a professional is more beneficial. |
| Post-discharge components | | |
| Evidence-based pharmacological support of NRT and e-cigarettes | Provide the participant with evidence-based pharmacological support. | Pharmacological support was identified as a promising BCT in terms of likely effectiveness and feasibility in our second systematic review. All effective interventions included pharmacological support. |
| Telephone support: 1 x call days 1 – 3 days post-discharge, and weekly thereafter | To provide personalised and tailored support to assist participants in achieving their behaviour change goals, and to provide feedback and encouragement on the progress of the participant’s individualised goals. Participant will receive tailored behavioural support via telephone or video call (dependent on preference). | Telephone support was used to increase motivation for smoking behaviour change (systematic review findings). However, frequency of timings varied. Evidence suggested relapse post-discharge is highest within the first 24 hours, only one study in our review offered telephone support within 24 hours post-discharge. BCTs in the ‘social support’ cluster were identified as promising in terms of likely effectiveness and feasibility in our second systematic review. |
| Unidirectional motivational texts alongside telephone support | To provide support to participants by texting tips to address tobacco norms encountered and challenges likely to be encountered, and to motivate participants by sending congratulatory texts when milestones have been reached. | Motivational text content well-received by participants in systematic review – offered practical and emotional support. BCTs in the ‘social support’ cluster were identified as promising in terms of likely effectiveness and feasibility in our second systematic review. |
| Identification and support of patient ‘buddy’ for cessation | To identify a family member/friend to offer additional support to the participant upon discharge. | Social support provided by healthcare professionals was frequently identified in existing evidence. Potential to use family/friends/carer as a buddy for additional support. |
| Helping healthcare professionals to support patients in changing their smoking behaviours | | |
| Specialist stop smoking training with mental health module, plus a clinician tailored top-up session | To ensure the mental health worker has successfully completed NCSCT training and specialist top-up modules. A training session in relation to study processes and procedures will also be conducted. | Specialist professional delivering the support package to complete NCSCT training and have additional sessions to focus on the benefits of supporting stopping smoking and links with mental health, and the safety and benefits of NRT and e-cigarettes. These sessions would be on-demand and available pre-recorded. |

## Table 2b. Feedback from key stakeholders and refinements to prototype intervention

|  | | |
| --- | --- | --- |
| Prototype intervention components | **Key stakeholder feedback** | **Refinement to prototype intervention** |
| Pre-discharge components | | |
| Pre-discharge assessment and flexible goal setting | This component was well-received by patients, mental healthcare workers and carers. All stakeholder’s believed personalisation was required to tap into individual’s motivations, wishes and needs. | No refinement required. |
| Patient resource pack provided by clinician at discharge | Patient stakeholders liked the idea of the pack for educational purposes, and suggested ideas for other resources to be added. These included success stories (to demonstrate positive behaviour changes of others), alternative activities for distraction purposes and financial trackers. Patient stakeholders also suggested some resources should be optional rather than compulsory (e.g., inclusion of smokefree home information). | Addition of resources including success stories, financial trackers, and lists of alternative activities for distraction purposes. Therefore, BCT of ‘distraction’ was identified as appropriate and met the APEASE criteria.  Made certain resources opt-in/opt-out, e.g., smokefree homes may not be appropriate for those in certain types of accommodation. |
|  | The resource pack was well received by mental healthcare workers. They believed relevant information from the pre-discharge evaluation session should be included in this folder for a smooth transition into the community (and those working in the community would be able to access this information without repeating assessments with participants).  Ideas for resources were well-received. Mental healthcare workers suggested these should be created in a user-friendly, creative way that is easy to read. | Include in the manual that information from the pre-discharge assessment is copied into the patient resource pack before discharge.  Resources to be created in a user-friendly way, in close collaboration with PPI group. |
| Post-discharge components | | |
| Evidence-based pharmacological support of NRT and e-cigarettes | Patient stakeholders suggested pharmacological support is beneficial, but they would have also preferred more advice and support around pharmacological support that is available, and how to obtain a longer-term supply in the future. | Pre-discharge assessment to provide advice on pharmacological support and how to obtain a longer-term supply. |
|  | Mental healthcare workers identified specialist training in e-cigarettes is required for the facilitator to offer any support around e-cigarettes. | No refinement required – e-cigarette training has been included in the prototype intervention. |
| Telephone support: 1 x call days 1 – 3 days post-discharge, and weekly thereafter | All key stakeholders highlighted the benefit of the telephone support calls and noted this was an important and essential component that would work particularly well if the support was individualised and tailored. Patient stakeholders highlighted they would want the same facilitator delivering the support throughout to develop a relationship. | The facilitator who starts the support with the participant at the pre-discharge evaluation session will continue offering all intervention components throughout the intervention period to ensure familiarity and allow a relationship to develop. |
| Unidirectional motivational texts alongside telephone support | All stakeholders believed this component was a good idea but suggested an opt-out system for those who did not want to receive text messages. Some patient stakeholders believed some may find compulsory text messages quite intrusive. | Change the text messaging to an opt-in/opt-out process that can be discussed during the pre-discharge evaluation session. |
| Identification and support of patient ‘buddy’ for cessation | All stakeholders believed the identification and support of a patient ‘buddy’ would not work. All stakeholders highlighted that a number of patients may not be able to identify someone to be their ‘buddy’, and if an individual is identified, they may not be willing or interested. All key stakeholders suggested an alternative of offering a peer interaction group to offer additional social support, if required. | Identification and support of a ‘patient’ buddy to be removed as a prototype intervention component and replace with an optional opportunity for peer interaction. This will be an informal, supportive environment that is primarily participant-led, but facilitated by a mental healthcare professional to encourage open and honest dialogue. |
|  | Patient stakeholders suggested peer support groups would offer help to those experiencing similar situations, which would also have a positive impact on mental health. A number of patient stakeholders suggested this group should be facilitated by a mental healthcare worker. |  |
| Helping healthcare professionals to support patients in changing their smoking behaviours | | |
| Specialist stop smoking training with mental health module, plus a clinician tailored top-up session | Patient stakeholders agreed this was an essential component and identified the need for the facilitator to have experience in smoking cessation and a mental health context. Patient stakeholders had no preference in relation to the job role of the individual delivering the support package. They wanted to ensure the facilitator had core traits such as compassion, patience and empathy, and the ability to communicate well with the participant. | Specialist professional is not necessarily required to deliver the support package if they encompass key traits and characteristics, and training is delivered.  Training to be delivered live, in an interactive manner with opportunities for questions and answers. |
|  | Mental health workers believed a specialist job role was not required if training was in place, and the facilitator had a positive attitude towards smoking cessation and understand the benefits of quitting smoking. Mental health workers believed the sessions would be beneficial, but it would be better to deliver them in a user-friendly and interactive format. |  |

## Table 2c. Feedback from expert group and further refinements to prototype intervention

| Prototype intervention components | Key stakeholder feedback | Refinement to prototype intervention |
| --- | --- | --- |
| Pre-discharge components | | |
| Pre-discharge assessment and flexible goal setting | Clinicians from participating Trusts suggested that this will need to include participants at all stages of smoking behaviour change. This will include those who want to (1) remain smokefree following abstinence/successful quit attempt during their hospital stay; (2) undertake a cessation attempt, or (3) cut down to quit/reduce to quit.  Clinicians from participating Trusts suggested the importance of the BCTs ‘pros and cons’ and ‘framing/reframing’ within a mental health inpatient setting. | The intervention manual will now include different pathways dependent on participant preference (cessation attempt or cut down/reduce to quit).  ‘Pros and cons’ and ‘framing/reframing’ will now be included as BCTs in the pre-discharge assessment and evaluation session to help the patient reflect on and evaluate their smokefree experience and explore/build motivation to remain abstinent or positively change their smoking behaviour. |
| Patient resource pack provided by clinician at discharge | Both clinicians and academic experts thought the patient resource pack (with additional resources as suggested by the key stakeholders) would be beneficial. | No further refinements made. |
| Post-discharge components | | |
| Evidence-based pharmacological support of NRT and e-cigarettes | Academic experts suggested that individuals may not want nicotine-based therapies. Varenicline would be acceptable if offered as part of usual care. | A range of evidence-based pharmacological support to be offered - including all options that are offered as part of usual care. No additional pharmacotherapies offered as part of the support package. |
| Telephone support: 1 x call days 1 – 3 days post-discharge, and weekly thereafter | Both clinicians and academic experts thought the telephone support would be beneficial. They agreed it would be advantageous for the same person to deliver the support throughout. | No refinement required. |
| Unidirectional motivational texts alongside telephone support | Academics and clinicians understood the decision to make the text messaging component opt-in/opt-out. However, they highlighted this has been found to be an effective component. Therefore, it would be more beneficial to begin by sending text messages to all participants with an opt-out option for participants if they do not wish to receive the text messages (by texting ‘STOP’). | Make the text messaging an opt-out component, whereby all participants are automatically enrolled and have the option to opt-out if they wish to do so. |
| Optional opportunity for peer support | Both academics and clinicians believed the addition of an optional remote peer engagement session would add value to the intervention. They agreed this should be moderated by a facilitator and suggested two staff members co-facilitate this group to offer support to one another. | The optional opportunity for peer support will be moderated by two facilitators. |
| Helping healthcare professionals to support patients in changing their smoking behaviours | | |
| Specialist stop smoking training with mental health module, plus a clinician tailored top-up session | Academics and clinicians agreed with the key stakeholders that it is not necessarily about the band or title of the specialist, but rather those who fulfil a certain criterion. For example, those who have experience in working with mental health and smoking cessation. The academics indicated there is no evidence to suggest that more qualified people are necessarily better in terms of their effectiveness in helping people to quit. Therefore, there is no strong reason to prefer trained nurses over healthcare assistants. Both clinicians and academics expressed some Trusts will have certain roles that other Trusts do not, so it would be unadvisable to exclude potential specialists due to their job/band, when they may be experienced and willing to deliver the intervention.  The training component was well received by both academics and clinicians and agreed with the stakeholders that the interactive nature of the bespoke training is important, particularly to provide staff members an opportunity to ask questions during the training and participate in role play activities. | No further refinement. |

| Supplementary Material 3. Findings from the behavioural analysis mapped onto the BCW. ^[[1]](#footnote-1)^ **indicates ‘promising’ BCTs in terms of probable effectiveness; **indicates ‘promising’ BCTs in terms of probable effectiveness and feasibility.* | | | | | |
| --- | --- | --- | --- | --- | --- |
| What needs addressing in the intervention based on literature | **Relevant TDF** | **COM-B** | **Intervention functions** | **Policy categories** | **BCTs linked to intervention functions** |
| Mental health professionals lack knowledge regarding smoking cessation support, policies, and lack knowledge regarding smoking in a mental health context, leading to the provision of misinformation. | Knowledge | Psychological capability | Education  Training  Enablement | Communication/marketing  Guidelines  Regulation  Legislation  Fiscal measures  Environmental/social planning  Service provision | - 1. Goal setting (behaviour)**   1.2 Problem solving**   - 1. Goal setting (outcome)   2. Action planning**   3. Review behaviour goal(s)   1.7 Review outcome goal(s)  2.2 Feedback on behaviour*  2.3 Self-monitoring of behaviour  2.7 Feedback on outcome(s) of behaviour  3.1 Social support (unspecified)**  3.2 Social support (practical)**  4.1 Instruction on how to perform the behaviour**  5.1 Information about health consequences**  5.3 Information about social and environmental consequences  6.1 Demonstration of the behaviour  7.1 Prompts/cues  8.1 Behavioural practice/rehearsal  12.1 Restructuring the physical environment  12.5 Adding objects to the environment |
| The need for structured patient education to enhance knowledge and awareness of the benefits of quitting smoking and links with mental health. | Knowledge | Psychological capability | Education  Training  Enablement | Communication/marketing  Guidelines  Regulation  Legislation  Fiscal measures  Environmental/social planning  Service provision | 1.1 Goal setting (behaviour)**   - 1. Problem solving**   2. Goal setting (outcome)   3. Action planning**   1.5 Review behaviour goal(s)  1.7 Review outcome goal(s)  2.2 Feedback on behaviour*  2.3 Self-monitoring of behaviour  2.7 Feedback on outcome(s) of behaviour  3.1 Social support (unspecified)**  3.2 Social support (practical)**  4.1 Instruction on how to perform the behaviour**  5.1 Information about health consequences**  5.3 Information about social and environmental consequences  6.1 Demonstration of the behaviour  7.1 Prompts/cues  8.1 Behavioural practice/rehearsal  12.1 Restructuring the physical environment  12.5 Adding objects to the environment |
| Disjointed pathways between inpatient services and community – a coordinated approach to support is required. | Environmental context and resources | Physical opportunity | Training  Restriction  Environmental restructuring  Enablement | Guidelines  Fiscal measures  Regulation  Legislation  Service provision  Environmental/social planning | - 1. Goal setting (behaviour)**   2. Problem solving**   3. Goal setting (outcome)   1.4 Action planning**  1.5 Review behaviour goal(s)  1.7 Review outcome goal(s)  2.2 Feedback on behaviour*  2.7 Feedback on outcome(s) of behaviour  2.3 Self-monitoring of behaviour  3.1 Social support (unspecified)**  3.2 Social support (practical)**  4.1 Instruction on how to perform the behaviour**  6.1 Demonstration of the behaviour  7.1 Prompts/cues  8.1 Behavioural practice/rehearsal  12.1 Restructuring the physical environment  12.5 Adding objects to the environment |
| Limited time to support patients and requirement to prioritise conflicting demands – identification of an appropriate person is required. | Environmental context and resources | Physical opportunity | Training  Restriction  Environmental restructuring  Enablement | Guidelines  Fiscal measures  Regulation  Legislation  Service provision  Environmental/social planning | - 1. Goal setting (behaviour)**   2. Problem solving**   3. Goal setting (outcome)   1.4 Action planning**  1.5 Review behaviour goal(s)  1.7 Review outcome goal(s)  2.2 Feedback on behaviour*  2.7 Feedback on outcome(s) of behaviour  2.3 Self-monitoring of behaviour  3.1 Social support (unspecified)**  3.2 Social support (practical)**  4.1 Instruction on how to perform the behaviour**  6.1 Demonstration of the behaviour  7.1 Prompts/cues  8.1 Behavioural practice/rehearsal  12.1 Restructuring the physical environment  12.5 Adding objects to the environment |
| Influence of social network members included social network members who smoked, smoking with network members, network members enabling smoking behaviours, and the impact of smoking norms, attitudes, and behaviours of social network members. | Social influences | Social opportunity | Restriction  Environmental restructuring  Modelling  Enablement | Guidelines  Regulation  Legislation  Fiscal measures  Environmental/social planning  Communication/marketing  Service provision | - 1. Goal setting (behaviour)**   2. Problem solving**   1.3 Goal setting (outcome)  1.4 Action planning**  1.5 Review behaviour goal(s)  1.7 Review outcome goal(s)  2.3 Self-monitoring of behaviour  3.1 Social support (unspecified)**  3.2 Social support (practical)**  6.1 Demonstration of the behaviour  7.1 Prompts/cues  12.1 Restructuring the physical environment  12.5 Adding objects to the environment |
| Limited self-efficacy and perceived ability to quit smoking (both patients and mental health professionals). | Beliefs about capabilities | Reflective motivation | Education  Persuasion  Modelling  Enablement | Communication/marketing  Guidelines  Regulation  Legislation  Service provision  Fiscal measures  Environmental/social planning | - 1. Goal setting (behaviour)**   2. Problem solving**   1.3 Goal setting (outcome)  1.4 Action planning**  1.5 Review behaviour goal(s)  1.7 Review outcome goal(s)  2.2 Feedback on behaviour*  2.3 Self-monitoring of behaviour  2.7 Feedback on outcome(s) of behaviour  3.1 Social support (unspecified)**  3.2 Social support (practical)**  5.1 Information about health consequences**  5.3 Information about social and environmental consequences  6.1 Demonstration of the behaviour  7.1 Prompts/cues  9.1 Credible source  12.1 Restructuring the physical environment  12.5 Adding objects to the environment |
| Expectations of abstinence and/or making a quit attempt – knowing what to expect, what support is offered and how they would be assisted by mental health professionals. | Beliefs about consequences | Reflective motivation | Education  Persuasion  Modelling | Communication/marketing  Guidelines  Regulation  Legislation  Service provision | 2.2 Feedback on behaviour*  2.3 Self-monitoring of behaviour  2.7 Feedback on outcome(s) of behaviour  5.1 Information about health consequences**  5.3 Information about social and environmental consequences  6.1 Demonstration of the behaviour  7.1 Prompts/cues  9.1 Credible source |
| Mental health professionals may not hold the positive intentions to support smoking cessation, and do not intend to recommend, advise, or demonstrate use of pharmacological support to aid smoking cessation. | Intentions | Reflective motivation | Education  Persuasion  Incentivisation  Coercion  Modelling | Communication/marketing  Guidelines  Regulation  Legislation  Service provision  Fiscal measures | 2.1 Monitoring of behaviour by others without feedback  2.2 Feedback on behaviour*  2.3 Self-monitoring of behaviour  2.5 Monitoring of outcome(s) of behaviour without feedback  2.7 Feedback on outcome(s) of behaviour  5.1 Information about health consequences**  5.3 Information about social and environmental consequences  6.1 Demonstration of the behaviour  7.1 Prompts/cues  9.1 Credible source |
| Patients need support to maintain a positive attitude while making a quit attempt. | Optimism | Reflective motivation | Education  Persuasion  Modelling  Enablement | Communication/marketing  Guidelines  Regulation  Legislation  Service provision  Fiscal measures  Environmental/social planning | - 1. Goal setting (behaviour)**   2. Problem solving**   1.3 Goal setting (outcome)  1.4 Action planning**  1.5 Review behaviour goal(s)  1.7 Review outcome goal(s)  2.2 Feedback on behaviour*  2.3 Self-monitoring of behaviour  2.7 Feedback on outcome(s) of behaviour  3.1 Social support (unspecified)**  3.2 Social support (practical)**  5.1 Information about health consequences**  5.3 Information about social and environmental consequences  6.1 Demonstration of the behaviour  7.1 Prompts/cues  9.1 Credible source  12.1 Restructuring the physical environment  12.5 Adding objects to the environment |
| The culture of perception that mental health professionals often view tobacco as a medical issue, not within a mental health provider’s scope of practice. | Professional/  social role and identity | Reflective motivation | Education  Persuasion  Modelling | Communication/marketing  Guidelines  Regulation  Legislation  Service provision | 2.2 Feedback on behaviour*  2.3 Self-monitoring of behaviour  2.7 Feedback on outcome(s) of behaviour  5.1 Information about health consequences**  5.3 Information about social and environmental consequences  6.1 Demonstration of the behaviour  7.1 Prompts/cues  9.1 Credible source |
| Personalised goals need to be set for each participant, including regular review to ensure appropriateness and provide feedback on whether the goals are being obtained. | Goals | Reflective motivation | Education  Persuasion  Incentivisation  Coercion  Modelling  Enablement | Communication/marketing  Guidelines  Regulation  Legislation  Service provision  Fiscal measures  Environmental/social planning | - 1. Goal setting (behaviour)**   2. Problem solving**   1.3 Goal setting (outcome)  1.4 Action planning**  1.5 Review behaviour goal(s)  1.7 Review outcome goal(s)  2.1 Monitoring of behaviour by others without feedback  2.2 Feedback on behaviour*  2.3 Self-monitoring of behaviour  2.5 Monitoring of outcome(s) of behaviour without feedback  2.7 Feedback on outcome(s) of behaviour  3.1 Social support (unspecified)**  3.2 Social support (practical)**  5.1 Information about health consequences**  5.3 Information about social and environmental consequences  6.1 Demonstration of the behaviour  7.1 Prompts/cues  9.1 Credible source  12.1 Restructuring the physical environment  12.5 Adding objects to the environment |
| Identifying personal motivators or incentives for quitting smoking and/or making progress with smoking-related behavioural goals (e.g., health or financial benefits). | Reinforcement | Automatic motivation | Training  Incentivisation  Coercion  Environmental restructuring | Guidelines  Fiscal measures  Regulation  Legislation  Service provision  Communication/marketing  Environmental/social planning | 4.1 Instruction on how to perform the behaviour**  6.1 Demonstration of the behaviour  2.1 Monitoring of behaviour by others without feedback  2.2 Feedback on behaviour*  2.3 Self-monitoring of behaviour  2.5 Monitoring of outcome(s) of behaviour without feedback  2.7 Feedback on outcome(s) of behaviour  7.1 Prompts/cues  8.1 Behavioural practice/rehearsal  12.1 Restructuring the physical environment  12.5 Adding objects to the environment |
| Addressing smoking as a coping mechanism for stress. Relapses in the context of acute stressors, smoking used to cope with everyday stresses and role as a coping mechanism in relation to mental health. | Emotion | Automatic motivation | Persuasion  Incentivisation  Coercion  Modelling  Enablement | Communication/marketing  Guidelines  Regulation  Legislation  Service provision  Fiscal measures  Environmental/social planning | - 1. Goal setting (behaviour)**   2. Problem solving**   1.3 Goal setting (outcome)  1.4 Action planning**  1.5 Review behaviour goal(s)  1.7 Review outcome goal(s)  2.1 Monitoring of behaviour by others without feedback  2.2 Feedback on behaviour*  2.3 Self-monitoring of behaviour  2.5 Monitoring of outcome(s) of behaviour without feedback  2.7 Feedback on outcome(s) of behaviour  3.1 Social support (unspecified)**  3.2 Social support (practical)**  6.1 Demonstration of the behaviour  9.1 Credible source  12.1 Restructuring the physical environment  12.5 Adding objects to the environment |
| Boredom, inactivity, and filling time prompts individuals to smoke, and stress and boredom can challenge abstinence – distraction activities to be advised. | Emotion | Automatic motivation | Persuasion  Incentivisation  Coercion  Modelling  Enablement | Communication/marketing  Guidelines  Regulation  Legislation  Service provision  Fiscal measures  Environmental/social planning | - 1. Goal setting (behaviour)**   2. Problem solving**   1.3 Goal setting (outcome)  1.4 Action planning**  1.5 Review behaviour goal(s)  1.7 Review outcome goal(s)  2.1 Monitoring of behaviour by others without feedback  2.2 Feedback on behaviour*  2.3 Self-monitoring of behaviour  2.5 Monitoring of outcome(s) of behaviour without feedback  2.7 Feedback on outcome(s) of behaviour  3.1 Social support (unspecified)**  3.2 Social support (practical)**  6.1 Demonstration of the behaviour  9.1 Credible source  12.1 Restructuring the physical environment  12.5 Adding objects to the environment |

# **Supplementary Material 4**. APEASE criteria for intervention functions and policy categories

## Supplementary Table 4a. Selection of intervention functions

|  | |
| --- | --- |
| Candidate intervention function | **Does the intervention function meet the APEASE criteria?** |
| Education | Yes |
| Persuasion | No – not practicable and unlikely to be effective. |
| Incentivisation | No – not affordable, practicable or cost effective. |
| Coercion | No – not practicable or acceptable. |
| Training | Yes |
| Restriction | No – not practicable. |
| Environmental restructuring | No – not practicable. |
| Modelling | No – not practicable. |
| Enablement | Yes |

## Supplementary Table 4b. Selection of policy categories to support intervention delivery

| Policy category | Does the intervention function meet the APEASE criteria? |
| --- | --- |
| Communications/marketing | Yes |
| Guidelines | Yes |
| Fiscal measures | No – not affordable, practicable, cost effective or acceptable. |
| Regulation | No – not practicable or acceptable. |
| Legislation | No – not practicable. |
| Environmental/social planning | No – not practicable. |
| Service provision | Yes |

# **Supplementary Material 5.** Selected BCTs for the prototype intervention and their definition

|  | |
| --- | --- |
| BCT label | **BCT definition**  (Michie et al., 2014) |
| 1.1 Goal setting (behaviour) | Set or agree on a goal defined in terms of the behaviour to be achieved. |
| 1.2 Problem solving | Analyse, or prompt the person to analyse, factors influencing the behaviour and generate or select strategies that include overcoming barriers and/or increasing facilitators (includes ‘relapse prevention’ and ‘coping planning’). |
| 1.3 Goal setting (outcome) | Set or agree on a goal defined in terms of a positive outcome of wanted behaviour |
| 1.4 Action planning | Prompt detailed planning performance of the behaviour (must include at least one of context, frequency, duration, and intensity). Context may be environmental (physical or social) or internal (physical, emotional, or cognitive) |
| 1.5 Review behaviour goal(s) | Review behaviour goal(s) jointly with the person and consider modifying goal(s) or behaviour change strategy in light of achievement. This may lead to re-setting the same goal, a small change in that goal or setting a new goal instead of (or in addition to) the first, or no change. |
| 1.7 Review outcome goal(s) | Review outcome goal(s) jointly with the person and consider modifying goal(s) in light of achievement. This may lead to re-setting the same goal, a small change in that goal or setting a new goal instead of, or in addition to the first. |
| 2.2 Feedback on behaviour | Monitor and provide informative or evaluative feedback on performance of the behaviour (e.g., form, frequency, duration, intensity). |
| 2.3 Self-monitoring of behaviour | Establish a method for the person to monitor and record their behaviour(s) as part of a behaviour change strategy. |
| 3.1 Social support (unspecified) | Advise on, arrange of provide social support (e.g., from friends, relatives, colleagues, buddies, or staff) or non-contingent praise or reward for performance of the behaviour. It includes encouragement and counselling, but only when it is directed at the behaviour. |
| 3.2 Social support (practical) | Advise on, arrange, or provide practical help (e.g., from friends, relatives, colleagues, ‘buddies’ or staff) for performance of the behaviour. |
| 3.3 Social support (emotional) | Advise on, arrange, or provide emotional support (e.g., from friends, relatives, colleagues, ‘buddies’ or staff) for performance of the behaviour. |
| 5.1 Information about health consequences | Provide information (e.g., written, verbal, visual) about health consequences of performing the behaviour. |
| 5.3 Information about social and environmental consequences | Provide information (e.g., written, verbal, visual) about social and environmental consequences of performing the behaviour. |
| 7.1 Prompts/cues^1^ | Introduce or define environmental or social stimulus with the purpose of prompting or cueing the behaviour. The prompt or cue would normally occur at the time or place of performance. |
| 9.2 Pros and cons | Advise the person to identify and compare reasons for wanting (pros) and not wanting to (cons) change the behaviour |
| 10.4 Social reward | Arrange verbal or non-verbal reward if and only if there has been effort and/or progress in performing the behaviour. |
| 10.9 Self-reward | Prompt self-praise or self-reward if and only if there has been effort and/or progress in performing the behaviour. |
| 11.1 Pharmacological support | Provide, or encourage the use of or adherence to, drugs to facilitate behaviour change. |
| 12.1 Restructuring the physical environment | Change, or advise to change the physical environment to facilitate performance of the wanted behaviour or create barriers to the unwanted behaviour. |
| 12.2 Restructuring the social environment | Change, or advise to change the social environment to facilitate performance of the wanted behaviour or create barriers to the unwanted behaviour. |
| 12.4 Distraction^2^ | Advise or arrange to use an alternative focus for attention to avoid triggers for unwanted behaviour. |
| 13.2 Framing/reframing^1^ | Suggest the deliberate adoption of a perspective or new perspective on behaviour (e.g., its purpose) in order to change cognitions or emotions about performing the behaviour. |
| ^1^ The BCTs ‘pros and cons’ and ‘framing/reframing’ were identified following consultations with the expert advisory group; met the APEASE criteria, and were identified as ‘promising’ in terms of likely effectiveness ^11^.  ^2^The BCT ‘distraction’ was identified following stakeholder consultation, which also met the APEASE criteria. | |

| Supplementary Material 6. Prototype intervention strategy | | | |
| --- | --- | --- | --- |
| Intervention function | **COM-B components served by intervention functions** | **Policy categories through which BCTs can be delivered^[[2]](#footnote-2)^** | **Intervention strategy (BCTs delivered)** |
| Enablement | Psychological capability  Social opportunity  Automatic motivation | Service provision | The patient will receive a pre-discharge evaluation session prior to discharge to plan for continuation of abstinence or further smoking behaviour change. A trained mental health worker will meet with the participant to offer support (BCT - social support (unspecified)), and initiate discussion about participant plans for their smoking post-discharge. The patient will work with a mental health worker to set clear, personalised goals (BCT – goal setting (behaviour)) and goals for either stopping smoking or reducing smoking (BCT – goal setting (outcome)). The mental health worker will discuss pharmacological support, providing advice on how to obtain a longer-term supply (BCT – pharmacological support). The mental health worker will also encourage the patient to identify specific triggers that may generate their urge to smoke and develop strategies for avoiding certain triggers (BCT – problem solving). |
| Education | Psychological capability  Reflective motivation | Service provision  Communication/marketing | During the pre-discharge evaluation session, the mental health worker will provide the patient with a personalised folder to assist them with achieving their behaviour change goals. This will provide educational content such as information sheets on the health benefits of quitting smoking (BCT - information about health consequences) and information about environmental consequences such as secondhand smoking (BCT – information about social and environmental consequences). Materials will be included such as smokefree signs with the purpose of prompting the behaviour in the participants home or car (BCT – prompts/cues). The folder will also include motivational and pages to document progress. The participant will be encouraged to record their progress daily (BCT – self-monitoring of behaviour). |
| Enablement | Psychological capability  Social opportunity  Automatic motivation | Service provision | After discharge, the mental health worker will provide personalised and tailored support via telephone or video call to assist the participant in achieving their behavioural change goals. The mental health worker will offer emotional support (BCT - social support (emotional)), e.g., encourage the participant and provide motivation, and offer practical support (BCT – social support (practical)), e.g., how to obtain a longer-term supply of NRT (BCT – pharmacological support). |
| Enablement | Psychological capability  Social opportunity  Automatic motivation | Service provision | During the support calls, the mental health worker will review the goals initially set in the pre-discharge evaluation session to ensure they are being met and are still appropriate (BCTs – review behaviour goals; review outcome goals). If these are not being met, the mental health worker will work with the participant to set new goals or generate new strategies that may help to overcome the barriers associated with goals not being met (BCT – action planning). The mental health worker will provide feedback on the participant’s behaviour (BCT – feedback on behaviour) and discuss the individual’s progress in relation to their goals. |
| Enablement | Psychological capability  Social opportunity  Automatic motivation | Service provision | Participants will receive text messages that provide tips (e.g., addressing tobacco norms encountered and challenges likely to be encountered) (BCT – social support (practical)) and motivational content (e.g., motivating participants by sending congratulatory messages when milestones have been reached) (BCTs – social reward, social support (emotional)). Text messages will also encourage participants to restructure their social and physical environment to distract them from cravings. For example, ‘try to spend more time with people that support your decisions and avoid being around others who smoke’ (BCT – restructure social environment), and ‘environmental changes are important to help quit smoking – make a smokefree space, throw away cigarettes and ashtrays’ (BCT – restructure physical environment). Text messages will also encourage participants to reward themselves if they have adhered to their goals (BCT – self-reward). |
| Enablement | Psychological capability  Social opportunity  Automatic motivation | Service provision | The mental health worker will help the participant to identify a ‘buddy’ for the participant post-discharge, who will offer support and assistance with meeting their smoking-related goals (BCT – social support (emotional; practical; unspecified)). |

# **Supplementary Material 7.** Mental healthcare workers mean scores, medians, ranges, and interquartile ranges (IQRs) for each intervention component in terms of practicality, acceptability, and ease of integration.

|  | | | | |
| --- | --- | --- | --- | --- |
|  | **Practicality** | | | |
| Intervention components | **Mean score** | **Median** | **Range** | **IQR** |
| My Try kit | 4.5 | 5 | 3-5 | 1 |
| Pre-discharge assessment | 4.2 | 5 | 1-5 | 1 |
| Patient ‘buddy’ | 3.4 | 4 | 1-5 | 1 |
| Behavioural support via telephone | 4 | 5 | 1-5 | 2 |
| Motivational texts | 4.1 | 5 | 1-5 | 1.5 |
| Staff training | 4.2 | 5 | 1-5 | 1 |
|  | **Acceptability to staff** | | | |
| Intervention components | **Mean score** | **Median** | **Range** | **IQR** |
| My Try kit | 4.4 | 5 | 1-5 | 1 |
| Pre-discharge assessment | 4.2 | 5 | 2-5 | 1.5 |
| Patient ‘buddy’ | 3.9 | 4 | 2-5 | 2 |
| Behavioural support via telephone | 4.2 | 4 | 3-5 | 1 |
| Motivational texts | 4.5 | 5 | 3-5 | 1 |
| Staff training | 4.3 | 4 | 2-5 | 1 |
|  | **Acceptability to patients** | | | |
| Intervention components | **Mean score** | **Median** | **Range** | **IQR** |
| My Try kit | 4.6 | 5 | 3-5 | 1 |
| Pre-discharge assessment | 4.4 | 5 | 2-5 | 1 |
| Patient ‘buddy’ | 3.7 | 3 | 2-5 | 2 |
| Behavioural support via telephone | 4.4 | 4 | 3-5 | 1 |
| Motivational texts | 4.5 | 5 | 3-5 | 1 |
| Staff training | 4.5 | 5 | 2-5 | 1 |
|  | **Acceptability to carers** | | | |
| Intervention components | **Mean score** | **Median** | **Range** | **IQR** |
| My Try kit | 4.5 | 5 | 2-5 | 0 |
| Pre-discharge assessment | 4.8 | 5 | 3-5 | 0 |
| Patient ‘buddy’ | 4.6 | 5 | 3-5 | 1 |
| Behavioural support via telephone | 4.7 | 5 | 3-5 | 0 |
| Motivational texts | 4.7 | 5 | 2-5 | 0 |
| Staff training | 4.8 | 5 | 3-5 | 0 |
|  | **Ease of integration into existing services** | | | |
| Intervention components | **Mean score** | **Median** | **Range** | **IQR** |
| My Try kit | 4.5 | 5 | 3-5 | 1 |
| Pre-discharge assessment | 4.5 | 5 | 3-5 | 1 |
| Patient ‘buddy’ | 3.2 | 3 | 1-5 | 2 |
| Behavioural support via telephone | 3.8 | 4 | 2-5 | 2 |
| Motivational texts | 4 | 5 | 1-5 | 2 |
| Staff training | 4.2 | 4 | 2-5 | 1 |

| Supplementary Material 8. Example quotations for themes | | |
| --- | --- | --- |
| Theme | **Stakeholder** | **Quotation** |
| Enablers to patient engagement and intervention delivery | Mental health worker  *(community smoking cessation advisor)* | “Post-discharge support is particularly important as there are no restrictions in the community – the motivational process needs to already start on the inpatient ward, and this is why the resource pack is such a good idea. It will contribute towards a positive attitude, and really great to provide a plan to people before they are discharged to continue motivation to stop smoking/stay quit – then there is a real transition from inpatient to community and the journey is continued when they have been discharged”. |
|  | Mental health worker  *(community healthy living advisor)* | “At the moment, there is no link between the ward and the community. When the pre-discharge assessment has been done in the past, there is no link with the community, so it has to be re-done and re-done. This can be really stressful for the patient. The assessment is so important, but it must be fed into the community, so it’s not duplicated unnecessarily. It is a good idea to put it in the kit, so everything is in one place and creates a kind of continuum”. |
|  | Mental health worker  *(smoking cessation advisor)* | “Telephone support is a great idea as it is easy to contact people and they are reachable – I have found that many are reluctant to physically go to services despite being available, may be lack of motivation but may be cost of transport. Remote delivery may increase both access and motivation”. |
|  | Patient  *(community)* | “I really like the clear goal setting idea, we are all motivated by different things, whether we want to quit or cut down for health or money, etc., it is important this is considered. The journal is a good idea in the kit – like the blank pages that allow for individualisation given the fact motivations vary across service users” |
| Potential barriers to intervention delivery | Mental health worker  *(inpatient nurse)* | “You need to have someone with the dedicated time to be able to provide telephone support. Current time constraints are an issue, and the person who delivers the call needs to be 100% dedicated and not let it slip through the net. If the calls do not happen, it will really dishearten the individual and make it look like you don’t care, which can in fact be extremely detrimental”. |
|  | Mental health worker  *(inpatient healthcare assistant)* | “Staff awareness of the benefits of stopping smoking is key – the majority of staff are smokers, and it has been really difficult to convince them of the benefits, this is a huge barrier”. |
|  | Patient  *(community)* | “I did not get any support at all in hospital, and if I had done, I may have been able to quit. Staff members in hospital do not know how to deal with smoking because they all smoke themselves”. |
|  | Caregiver | “Training is a great idea for staff and something that absolutely needs to happen – it is essential to offer effective support to patients, but many staff members do not have the required training to offer this support, and you’ll find the majority are smokers as well”. |
| Overall perceptions | Mental health worker  *(community smoking cessation advisor)* | “The resource kit is a great idea – patients find it difficult to retain information so having everything required in a kit on discharge is innovative”. |
|  | Mental health worker  (*community healthy living advisor)* | “The kit would be well-received by patients – there is not a great deal of support in the community to give to people due to lack of resources. We only have posters in clinics which can be referred to, but much more is needed. Information is absolutely key and is really important to include”. |
|  | Mental health worker  *(inpatient nurse)* | “The goal setting and assessment session is a great idea and there is a huge need for goal setting and planning – this can easily be integrated into services as many have a checklist for things to do before discharge” |
|  | Patient  *(community)* | “The resource kit is a great idea. It provides information needed to people who may not know the benefits. I am still unaware of the complete health consequences now and why it may be advantageous to quit for my mental being, so this would have helped a lot”. |
|  | Patient  *(community)* | “A message first thing in the morning would be helpful in ‘setting the tone for the day and a reminder of the focus of what I was doing. One-way messages would be sufficient.” |
|  | Patient  *(community)* | “Telephone support is the best idea – speaking to somebody is really helpful whether it be face to face or over the phone. Speaking to somebody with more knowledge on how to control the smoking is really helpful for someone who doesn’t know how, particularly as I don’t know where to start with quit attempts and feel a lack of support in the hospital and in the community”. |
| Practical considerations and suggestions | Mental health worker  *(inpatient nurse)* | “Family support is absolutely key to cessation, and it is beneficial for people to have social support post-discharge, but a lot of people do not have that person, or do not have someone who is willing or interested – so it may be an idea to provide an opportunity for some kind of peer support”. |
|  | Mental health worker  *(community healthy living advisor)* | “The NCSCT training can be quite difficult to take in. If an extra top-up session was provided, it would be great if this was in a more user-friendly format, like an interactive session over Zoom or a recorded video”. |
|  | Patient  *(community)* | “It would be good to add success stories of quitting in the resource kit, this would be good to see what has worked for others in the past and would give me motivation” |
|  | Patient  *(community)* | “People may not have family who can assist. Could you offer a casual support group between service users where people can chat about how they’re getting on? The social time will also help with their mental health”. |
|  | Patient  *(community)* | “Would add some leaflets in about activities that may offer some distraction from smoking, like physical activity or arts and crafts – something that may tap into individual’s motivations to stop smoking. Many people want to quit but don’t know where to start or what will work for them, so including information on various activities that can be used for distraction may work for individual’s interests or preferences”. |
|  | Caregiver | “A support group may be a better way of promoting that required social support – by working together and stopping together, this may be better than identifying someone for support who may not necessarily have experience of trying to stop smoking or cutting down. It could be the ‘buddy’ is a smoker too, so this makes it even harder”. |

| Supplementary Material 9. Final prototype intervention mapping table | | | | | | |
| --- | --- | --- | --- | --- | --- | --- |
| Finding | **TDF domain** | **COM-B domain** | **Intervention function** | **Policy category** | **Selected BCTs** | **Proposed operationalisation of selected intervention components** |
| Mental health professionals lack knowledge regarding smoking in a mental health context | Knowledge | Psychological capability | Training | Service provision | 2.2 Feedback on behaviour  5.1 Information about health consequences  5.3 Information about social and environmental consequences | The My Try Specialist delivering the intervention will receive enhanced training and on-going support in supporting people with a mental health condition to change their smoking-related behaviours post-discharge. |
| Patients do not receive adequate education to enhance knowledge or awareness of the benefits of quitting smoking | Knowledge | Psychological capability | Education | Service provision  Communication/marketing | 2.3 Self-monitoring of behaviour  3.2 Social support (practical)  5.1 Information about health consequences  5.3 Information about social and environmental consequences  11.1 Pharmacological support | During the pre-discharge evaluation session, the My Try Specialist will provide the patient with a personalised folder to assist them with achieving their behaviour change goals. This will provide educational content such as information sheets on the health benefits of quitting smoking and information about environmental consequences such as secondhand smoking. The folder will also include motivational and pages to document progress. The participant will be encouraged to record their progress daily.  Practical support will also be provided during this session (e.g., how to obtain a longer-term supply of NRT once discharged). |
| Disjointed pathways between inpatient services and community | Environmental context and resources | Physical opportunity | Enablement | Guidelines  Service provision | 3.1 Social support (unspecified)  7.1 Prompts/cues | The patient will receive a pre-discharge evaluation session prior to discharge to plan for continuation of abstinence or further smoking behaviour change. The My Try Specialist will meet with the participant to offer support and initiate discussion about participant plans for their smoking post-discharge. These plans will be documented in the personalised resource folder, so the information is accessible in the community. |
| Limited time to support patients | Environmental context and resources | Physical opportunity | Enablement | Guidelines  Service provision | 12.1 Restructuring the physical environment | Introduce My Try Specialists who will be specifically identified and trained to deliver the support to patients, each with their own case load. |
| Mental health professionals may not hold the positive intentions to support smoking cessation | Intentions | Reflective motivation | Education | Service provision | 5.1 Information about health consequences  5.3 Information about social and environmental consequences | The My Try Specialists delivering the intervention will receive enhanced training and on-going support in supporting people with a mental health condition to change their smoking-related behaviours post-discharge. |
| Negative influence of social network members | Social influences | Social opportunity | Enablement | Service provision | 3.1 Social support (unspecified)  3.2 Social support (practical)  3.3 Social support (emotional)  12.2 Restructuring the social environment | An opportunity for peer interaction to provide patients with a forum to reflect on their experiences, share stories with each other, and derive social support from both the My Try Specialists and their peers.  Post-discharge text messages will also encourage participants to restructure their social and physical environment to distract them from cravings. For example, where possible, ‘try to spend more time with people that support your decisions and avoid being around others who smoke’. |
| Limited self-efficacy and perceived ability to quit smoking | Beliefs about capabilities | Reflective motivation | Enablement | Service provision | 3.3 Social support (emotional)  10.4 Social reward  10.9 Self-reward | After discharge, the My Try Specialist will provide personalised and tailored support via telephone or video call to assist the participant in achieving their behavioural change goals. The My Try Specialist will offer emotional support, encourage the patient, and provide motivation.  Additionally, patients will receive text message support to provide motivational content (e.g., congratulatory messages when milestones have been reached). Text messages will encourage participants to reward themselves if they have adhered to their goals. |
| Perception that mental health professionals view tobacco as a medical issue, not within their scope of practice | Professional/ social role and identity | Reflective motivation | Education | Service provision | 5.1 Information about health consequences  5.3 Information about social and environmental consequences | The My Try Specialist delivering the intervention will receive enhanced training and on-going support in supporting people with a mental health condition to change their smoking-related behaviours post-discharge. |
| Patients need support to maintain a positive attitude while making a quit attempt | Optimism | Reflective motivation | Enablement | Service provision | 3.3 Social support (emotional)  10.4 Social reward | After discharge, the My Try Specialist will provide personalised and tailored support via telephone or video call to assist the participant in achieving their behavioural change goals. The My Try Specialist will offer emotional support, encourage the patient, and provide motivation. |
| Personalised goals need to be set for each participant, including regular review to ensure appropriateness and provide feedback on whether the goals are being obtained | Goals | Reflective motivation | Enablement | Service provision | - 1. Goal setting (behaviour)   2. Goal setting   (outcome)  1.4 Action planning   - 1. Review behaviour goals   2. Review outcome   goals  2.2 Feedback on  Behaviour  9.2 Pros and cons  13.2 Framing/reframing | The patient will receive a pre-discharge reflection and evaluation session prior to discharge to plan for continuation of abstinence or further smoking behaviour change. The My Try Specialist will meet with the participant to discuss participant plans for their smoking post-discharge. The patient will work with the My Try Specialist to set clear, personalised goals and goals for either stopping smoking or reducing smoking.  Advice will also be provided during this session to help the patient identify and compare reasons for wanting (pros) and not wanting to (cons) change their behaviour. The My Try Specialist will offer different perspectives to current belief barriers and discuss self-management strategies in order to achieve their goals.  During the support calls, the My Try Specialist will review the goals set in the pre-discharge evaluation session to explore if they are being met and are still appropriate. Where appropriate, the MTS will work with the participant to set new goals or generate new strategies that may help to overcome the barriers associated with goals not being met. The My Try Specialist will provide feedback on the participant’s behaviour and discuss the individual’s progress in relation to their goals. |
| Addressing smoking as a perceived coping mechanism for stress | Emotion | Automatic motivation | Enablement | Service provision | - 1. Problem solving   5.1 Information about health consequences | The My Try Specialist will provide information to address the myth that smoking reduces stress. During the pre-discharge evaluation session and intervention, the My Try Specialist will encourage the patient to identify triggers that may generate their urge to smoke and develop strategies for avoiding or managing triggers. |
| Boredom, inactivity, and filling time prompts individuals to smoke | Emotion | Automatic motivation | Enablement | Service provision  Communication/marketing | 12.1 Restructuring the physical environment  12.2 Restructuring the social environment  12.4 Distraction | Additional resources are available to include in the personalised resource folder, including content that outlines alternative activities that may offer a distraction from smoking. This includes quotes from others who have experienced similar situations and how the activities have helped when changing their smoking-related behaviour. An activity planner is also available where patients can schedule their activities for the upcoming week. |

# **Supplementary Material 10.** Prototype intervention specification

**Pre-discharge reflection and evaluation**

Session(s) with My Try Specialist before discharge.

**Aim**

To provide personalised support to assist participants in achieving their goals.

**BCTs**

Goal setting (behaviour), goal setting (outcome), problem solving, action planning, social support (unspecified; emotional; practical), information about health consequences, pros and cons, framing/reframing, pharmacological support.


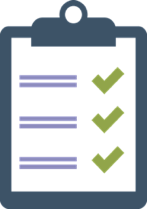


**Personalised resource folder (My Try)**

Provision of resource folder pre-discharge.

**Aim**

To provide participant with a personalised folder to assist them with achieving their goals. Resources to include motivational and practical content.

**BCTs**

Action planning, self-monitoring of behaviour, social support (practical; emotional), information about health consequences, information about social and environmental consequences, prompts/cues, distraction.


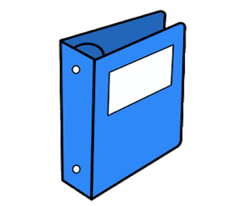


**Tailored behavioural support**

Sessions with My Try Specialist on days 1-3 post-discharge, and weekly thereafter (12 weeks).

**Aim**

To provide personalised support and encouragement to assist participant in achieving their goals, and to provide feedback and encouragement on progress.

**BCTs**

Problem solving, action planning, review behaviour goals, review outcome goals, feedback on behaviour, social support (practical; emotional), information about health consequences, pharmacological support.


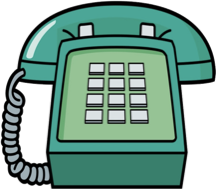

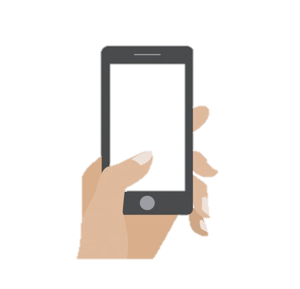


**Optional text-based support**

Semi-tailored motivational text messages from My Try Specialist.

**Aim**

To provide support to participants by texting practical tips and motivational content.

**BCTs**

Social support (unspecified; emotional; practical), problem solving, information about health consequences, self-reward, social reward, restructure social environment, restructure physical environment.

**Optional opportunity for peer interaction**

Monthly sessions run by two My Try Specialists.

**Aim**

To provide participants with a forum to reflect on their experiences and share stories with each other.

**BCTs**

Social support (unspecified; emotional; practical), problem solving.


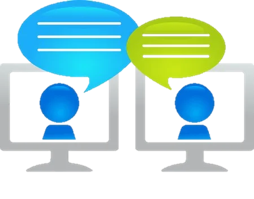


**Prototype Intervention Specification**

| Supplementary Material 11. Description of the prototype intervention using the TIDieR checklist ** Items 10 and 12 not included as these items are not relevant to the prototype intervention and cannot be described until the intervention is delivered.* | |
| --- | --- |
| TIDieR Checklist | **Description** |
| Item 1: Brief name | The intervention development process in this paper is part of the wider ‘SCEPTRE’ (Promoting **S**moking **CE**ssation and **P**reven**T**ing **RE**lapse to tobacco use following a smokefree mental health inpatient stay) programme. Therefore, the brief name is the ‘SCEPTRE intervention’. |
| Item 2: Why | The lack of support offered post-discharge and the high risk of relapse or return to pre-admission smoking patterns renders smoking-related resource input during the inpatient episode inefficient, as positive smoking behaviour change achieved during the inpatient stay is usually lost. Identifying interventions that are effective in supporting patients around the time of hospital discharge in maintaining or achieving positive change to their smoking behaviours has been identified as an important evidence gap in this area. |
| Item 3: What materials | The mental health worker delivering the intervention will be provided with a standardised intervention manual (including session outlines for behavioural support and the text message content).  At discharge, the patients will be provided with a personalised resource folder (‘My Try resource folder’) including practical and motivational content. The My Try resource folder will also be provided to the participant. This includes resources on the benefits of stopping smoking, journal pages for reflection, and a copy of the participants behaviour change plan. Participants can also select a range of additional resources to include in their folder, including a list of activities that others have found useful, a financial tracker, a Nicotine Replacement Therapy or e-cigarette log to document type, strength, and frequency (if applicable), a fact sheet on passive smoking and tips to making smokefree cars and homes, and success stories provided by others in similar situations. |
| Item 4: What procedures | Prior to discharge, the My Try Specialist will meet with the participant to initiate reflection on their smokefree stay, and discussion of their smoking-related behaviour change goals, individual motivations, pharmacological support, and planning of strategies and techniques to help them to meet their goals post-discharge.  Once the participant has been discharged, the My Try Specialist will offer weekly behavioural support via telephone or video call. These sessions aim to provide personalised and tailored support, assist the participant in achieving their behaviour change goals, and provide feedback and encouragement on the participant’s progress. The support calls will also offer the opportunity for the participant to reflect on their own progress and discuss whether their goals are being met. Participants will also be encouraged to engage with the intervention between the weekly sessions. This process will be supported by the My Try resource kit provided prior to discharge.  The text-message support will provide motivational and practical information to participants. The content of the text messages will vary depending on the participant’s motivation to quit and will be both personalised and tailored to the patient, using the patient’s name and information obtained during the pre-discharge assessment (see example in Supplementary Table 12a and 12b). Optional group support sessions will aim to facilitate peer-to-peer support, providing participants with a forum to reflect on their experiences and share stories with each other. My Try Specialists will be present in these groups to support participants and answer any questions that may be raised. |
| Item 5: Who provided | The intervention will be delivered by a mental health worker (the ‘My Try Specialist’) who will receive enhanced training and ongoing support in supporting people with mental health conditions to remain smokefree following discharge, or, where they have not been abstinent during admission, to achieve positive change to their smoking-related behaviours. The enhanced training will be delivered by means of interactive workshops. All My Try Specialists will receive and be instructed to use a manual to deliver the intervention. Self-reflection will be encouraged and supervision from the research team and the Trust clinicians will also be provided. The term ‘My Try Specialist’ was suggested by our research programme PPI group and links to the name of the personalised resource folder provided to the participants prior to discharge (‘My Try folder’). |
| Item 6: How | Training sessions for mental health workers will be conducted in groups, remotely or face-to-face (dependent on preference and needs).  The pre-discharge reflection and evaluation will be held face-to-face prior to discharge. The intervention components delivered post-discharge will be conducted remotely. Behavioural support calls will be offered on a one-to-one basis via telephone or video call, and peer support groups will be conducted via video call. |
| Item 7: Where | The pre-discharge reflection and evaluation will be conducted on the ward prior to discharge. The intervention components delivered post-discharge will be conducted remotely, so participants will be receiving the support in the community. |
| Item 8: When and how much | The My Try Specialist will meet with the participant for up to two pre-discharge reflection and evaluation sessions, dependent on needs and preferences. Once discharged, participants will be offered up to 14 individual behavioural support sessions with a My Try Specialist delivered by telephone or video call. The first three sessions will be conducted on the first three days post-discharge, and then weekly thereafter. The sessions will each last between 30 and 60 minutes depending on the needs and preferences of the participant.  Participants will also receive semi-tailored, unidirectional text messages from the My Try Specialist. The frequency of the text messages will vary depending on the participant’s motivation to quit (Supplementary Material 10). Where possible, optional peer interaction group sessions will be provided by two My Try Specialists via video call. The sessions will be held once a month and last approximately 60 minutes. Intervention endpoint will be 12 weeks after the participant’s first session with the My Try Specialist. |
| Item 9: Tailoring | The behavioural support sessions will be tailored to the individual’s needs and preferences, based on the information collated from the pre-discharge reflection and evaluation sessions. The text messages will also be semi-tailored, where the frequency and content of the messages vary depending on the participant’s motivation to quit. |
| Item 11: How well | The prototype intervention will be delivered in a small-scale pilot study to test the intervention adherence. |

# **Supplementary Material 12.** Example text message content for participants

## Supplementary Table 12a. Text message content – Quit attempt example

| Time | Objective | Message content | |
| --- | --- | --- | --- |
|  |  | **Text 1** | **Text 2** |
| Day 1 | Introduction | *Hi (name), deciding to quit is a huge step. You should be proud of yourself.* | *We are going to send you a daily text message over the next 15 days to help you to make changes related to your use of cigarettes. To stop these messages, please reply ‘STOP’ to this text message.* |
| Day 2 | Reminder about the goal | *Hi (name), we are happy to hear you are interested in quitting.* | *Don’t forget your goal! [Insert goals]* |
| Day 3 | Provide information on risk of tobacco use/cessation challenges | *Hi (name), congratulations for being concerned about your health and trying to quit smoking!* | *Smoking is damaging to your physical health. Keep this in mind to help you quit* |
| Day 4 | Provide information on benefits from quitting | *Hi (name), do you know that people who quit smoking reduce their risk of death from heart attack in half after a year?* | *Improvements in physical activities as well as money savings are other advantages of quitting.* |
| Day 5 | Provide information on second-hand smoking | *Hi (name), people that do not smoke but share spaces at home or at work with smokers are 30% more likely to have lung cancer and 25% more likely to have a heart attack.* | *Congratulations on your decision to change your smoking behaviour, you are also helping people close to you to preserve their health.* |
| Day 6 | Increase self-efficacy (past quit attempts) | *Hi (name), you were able to go [insert number here: obtained from pre-discharge evaluation] days during your hospitalisation without smoking and [insert number here: obtained from pre-discharge evaluation] days in a previous quit attempt.* | *This is a great achievement! Remember what helped you to stay smokefree during that time. This can help you overcome any current difficulties.* |
| Day 7 | Feedback about confidence – increase self-efficacy | *Hi (name), it is great to know you’re confident in your ability to stay smokefree.* | *Avoid being around other smokers while they smoke. If that is not possible, practice saying ‘no, thank you’. Talk to former smokers and learn how they quit* |
| Day 8 | Relapse prevention and self-efficacy | *Hi (name), it may be hard not to smoke when you are anxious or are at home watching TV.* | *Other activities like reading or gardening can help. Try out those activities, especially during the first few days* |
| Day 8 | Relapse prevention and self-efficacy | *Hi (name), it may be hard not to smoke when you are anxious or are at home watching TV.* | *Other activities like reading or gardening can help. Try out those activities, especially during the first few days* |
| Day 9 | Relapse prevention and self-efficacy | *Hi (name), it may help to be smokefree inside the home. Keep in mind that this strategy may help a lot.* | *Changes during the first days are really helpful!* |
| Day 10 | Provide information about social support | *Hi (name), telling close friends or relatives that you are quitting can be very helpful.* | *Try to spend more time with people that support your decisions and who can help distract you from cravings* |
| Day 11 | Provide information about environment changes | *Hi (name), environmental changes are important to help you quit smoking.* | *Make a smokefree space, throw away cigarettes and ashtrays.* |
| Day 12 | Reminder about reward for quitting | *Hi (name), what about saving the money that you spend on cigarettes to buy something for yourself?* | *You can use this money to buy something you want, or buy something for someone special, or even keep it in your savings account* |
| Day 13 | Provide information on withdrawal symptoms | *Hi (name), do not worry if you are craving a cigarette – this is normal.* | *Think about the most difficult moments and make a list of things that could help you keep your hands and mind busy.* |
| Day 14 | Provide information on withdrawal symptoms | *Hi (name), do you still crave a cigarette? Do not worry. Keep your hands and mind busy.* | *We are confident you can make it!* |
| Day 15 | Conclusion | *Hi (name), congratulations on getting this far!* | *This is the last text we will send now, but remember you will still receive your weekly calls. Congratulations again!* |

## Supplementary Table 12b. Example text message content – Not yet ready to quit example

| Time | Objective | Message content | |
| --- | --- | --- | --- |
|  |  | **Text 1** | **Text 2** |
| Day 1 | Introduction | *Hi (name), do not worry that you are not ready to quit just yet.*  *.* | *We are going to send you a daily text message over the next 8 days to help you to make changes related to your use of cigarettes. To stop these messages, please reply ‘STOP’ to this text message* |
| Day 2 | Orientation about risk of tobacco use | *Hi (name), congratulations for being concerned about your health and trying to change your smoking-related behaviours.* | *Smoking is damaging to your physical health. Keep this in mind to help you cut down* |
| Day 3 | Orientation about benefits from quitting | *Hi (name), do you know that people who quit smoking reduce their risk of death from heart attack in half after a year? .* | *Improvements in physical activities as well as money savings are other advantages of quitting* |
| Day 4 | Orientation about second hand smoking | *Hi (name), people that don’t smoke but share spaces at home or at work with smokers are 30% more likely to have lung cancer and 25% more likely to have a heart attack.* | *If you decide to change your smoking behaviour, you are also helping people close to you to preserve their health* |
| Day 5 | Orientation about financial reward | *Hi (name), what about saving the money that you spend on cigarettes to buy something for yourself?* | *You can use this money to buy something you want, or buy something for someone special, or even keep it in your savings account* |
| Day 6 | Reminder about tips to replace tobacco use | *Hi (name), it may be hard to plan to quit when you are feeling anxious.* | *Other activities like reading or gardening can help. Try out these activities, they may help to keep your mind and hands busy.* |
| Day 7 | Increase self-efficacy (past quit attempts) | *Hi (name), you were able to go [insert number here: obtained from pre-discharge evaluation] days during your hospitalization without smoking and [insert number here: obtained from pre-discharge evaluation] days in a previous quit attempt.* | *This is a great achievement! You could do this again!* |
| Day 8 | Conclusion | *Hi (name), we hope these texts have helped to build your confidence in changing your smoking-related behaviours.* | *This is the last text we will send now but remember you will still receive your weekly calls.* |

1. In our second systematic review (Shoesmith et al., 2021), BCTs were defined as ‘promising’ in terms of probable effectiveness if the technique was present in at least two long-term effective interventions, defined as those reporting statistically significant differences in smoking abstinence between intervention and control groups at a 6-month follow-up point or later. BCTs were also defined as ‘promising’ in terms of feasibility if they were also delivered in ≥ 25% interventions. [↑](#footnote-ref-1)
2. In Step 6, although training was identified as a potentially useful policy category, the intervention strategy shown here is to be delivered to patients. The intervention function training will be used for the delivery of specialist training to the facilitators delivering the intervention. [↑](#footnote-ref-2)
